# Extracellular Matrix Signaling Cues: Biological Functions, Diseases, and Therapeutic Targets

**DOI:** 10.1002/mco2.70281

**Published:** 2025-07-17

**Authors:** Tian Zhao, Ye Huang, Jingfei Zhu, Yujie Qin, Hao Wu, Jiaxuan Yu, Qianwen Zhai, Shun Li, Xiang Qin, Dengfeng Wang, Tingting Li, Yiyao Liu

**Affiliations:** ^1^ Department of Gynecologic Oncology Sichuan Clinical Research Center for Cancer Sichuan Cancer Hospital & Institute, Sichuan Cancer Center, and School of Life Science and Technology, University of Electronic Science and Technology of China Chengdu P. R. China; ^2^ TCM Regulating Metabolic Diseases Key Laboratory of Sichuan Province Hospital of Chengdu University of Traditional Chinese Medicine Chengdu P. R. China; ^3^ Department of Clinical Medicine North Sichuan Medical College Nanchong P.R. China; ^4^ Department of Urology Deyang People's Hospital Deyang P. R. China

**Keywords:** ECM dysregulation, ECM remodeling, ECM targeted therapies, extracellular matrix (ECM), mechanotransduction

## Abstract

Extracellular matrix (ECM) is a dynamic, three‐dimensional network that provides structural support and regulates key biological processes, including cell adhesion, migration, differentiation, and signal transduction. Its mechanical properties, such as stiffness, topology, and viscoelasticity, are crucial in normal and pathological conditions, influencing cell behavior through mechanotransduction pathways. Dysregulation of ECM is linked to various diseases, making a thorough understanding of its composition and properties essential. This review discusses ECM composition, physical properties, and the limitations of in vitro ECM models. It highlights the role of ECM in tissue homeostasis, particularly in regulating cell behavior via mechanotransduction, focusing on force‐sensitive sensors like integrins, Piezo1, TRPV4, and YAP/TAZ. Additionally, the review explores ECM remodeling in cancer, fibrosis, and cardiovascular diseases, along with current therapeutic strategies targeting ECM components, such as nanotechnology‐based therapies, small molecule inhibitors, and CAF‐targeted therapies. Challenges and clinical applications of these therapies are also discussed. Finally, the review looks ahead to future research, emphasizing the integration of ECM‐targeted therapies in precision medicine and novel approaches to normalizing ECM composition and structure for therapeutic benefits. This review provides mechanobiological insights into therapeutic strategies targeting the ECM.

## Graphical abstract

Targeting ECM dysregulation: therapeutic strategies for disease intervention. Dysregulated ECM composition and biomechanics contribute to disease pathogenesis in cancer and fibrotic disorders. Physical modulation: Nanomedicine‐based strategies tune ECM physical properties (e.g., density, stiffness) to enhance drug penetration and therapeutic efficacy in the TME. Molecular targeting: Small‐molecule drugs selectively modify ECM components to induce angiogenesis, immune response, and tissue remodeling. Cellular reprogramming: Pharmacological intervention of CAF signaling pathways promotes functional normalization, thereby restoring ECM homeostasis.

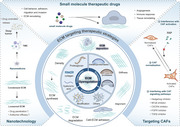

## 1 Introduction

The Extracellular matrix (ECM) is a highly dynamic and complex three‐dimensional network that provides not only structural support for tissues but also biochemical and mechanical cues essential for cellular function [1, 2]. Composed of macromolecules such as collagens, glycosaminoglycans, elastin, and proteoglycans [3], the ECM regulates fundamental biological processes, including cell adhesion, migration, differentiation, and signal transduction [4, 5]. Through these functions, the ECM plays a pivotal role in maintaining tissue homeostasis and orchestrating cellular responses to environmental stimuli [6].

Beyond its role in normal physiology, the mechanical properties of ECM, such as stiffness, topology, and viscoelasticity, serve as key regulators of cellular behavior via mechanotransduction pathways [4, 7]. Changes in ECM mechanics are frequently observed in pathological conditions, including cancer, fibrosis, and cardiovascular diseases, where dysregulated ECM remodeling promotes disease progression [8, 9, 10]. The aberrant stiffening of the ECM, for instance, enhances tumor invasion and fibrosis progression by altering cellular mechano‐signaling [11, 12, 13]. Understanding ECM composition, physical properties, and the molecular mechanisms governing its remodeling contributes to the development of effective therapeutic strategies.

Current advances in ECM‐targeted therapies offer promising strategies to mitigate disease‐associated ECM dysregulation. Approaches such as nanotechnology‐based ECM‐targeted delivery systems, small molecule inhibitors of ECM‐modulating enzymes, and cancer‐associated fibroblast (CAF)‐targeted therapies are under investigation for their potential to restore ECM homeostasis [14, 15, 16, 17]. However, challenges remain in optimizing these strategies for clinical application, particularly in enhancing specificity, minimizing off‐target effects, and achieving sustained therapeutic efficacy. For instance, CAF‐directed approaches struggle to reconcile the dual roles of CAFs in tumor suppression and promotion, while antifibrotic therapies face challenges in balancing ECM degradation with tissue integrity [18, 19].

In this review, we systematically explore the biological functions of ECM, its regulatory role in mechanotransduction via key pathways including integrins, piezo‐type mechanosensitive ion channel component 1 (Piezo1), transient receptor potential vanilloid 4 (TRPV4), and yes‐associated protein (YAP)/transcriptional coactivator with PDZ‐binding motif (TAZ), and its implications in disease pathogenesis. We critically evaluate current therapeutic approaches targeting ECM components and discuss their translational potential. Finally, we propose future perspectives for the integration of experimental models, drug development approaches, and innovative therapeutic strategies, providing biomechanical insights into the role of ECM‐targeted interventions in precision medicine.

## 2 Fundamental concepts of ECM biology

**FIGURE 1:**
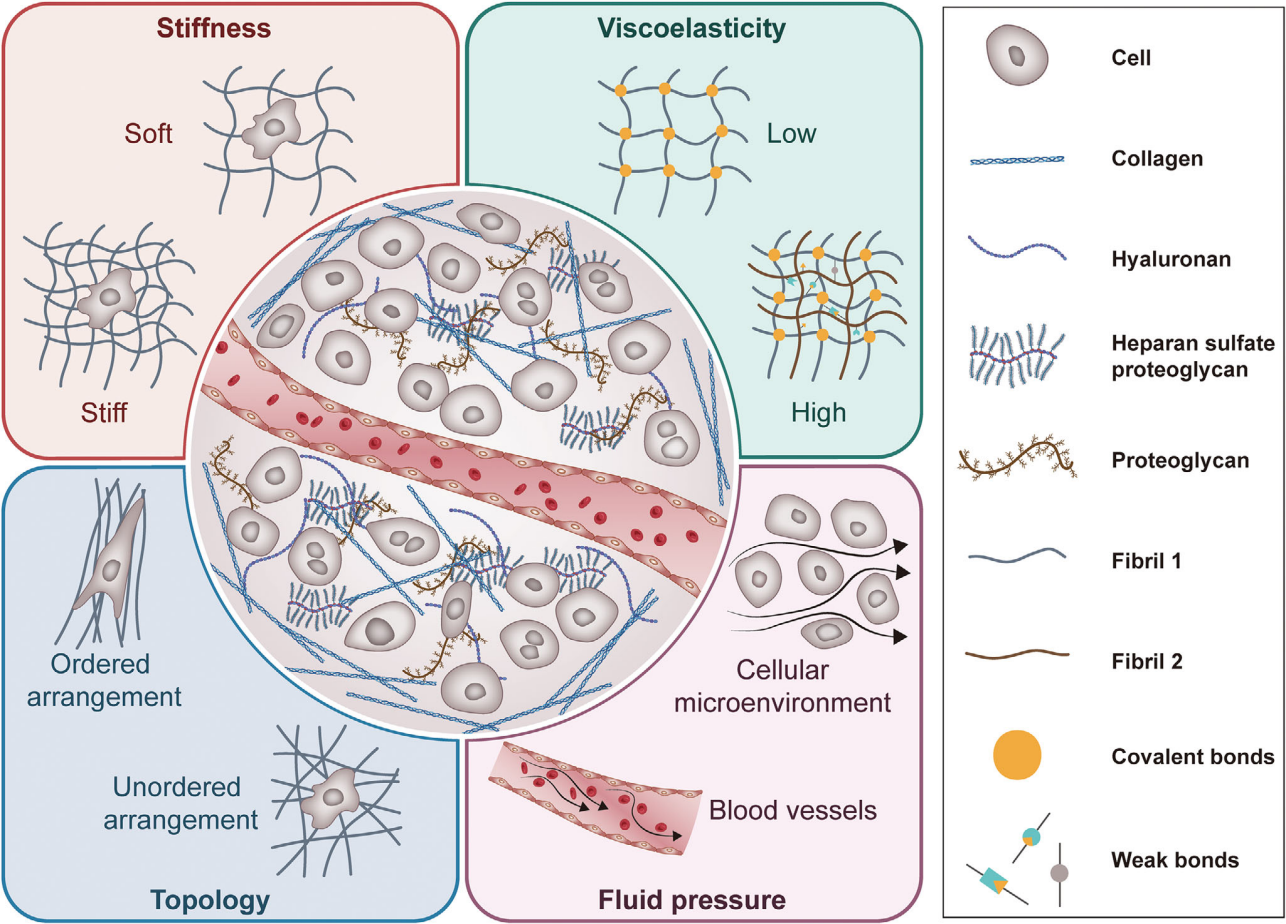
Physical properties of ECM. ECM consists of collagen, elastic fibers, glycoproteins, and proteoglycans. The ECM exhibits the following mechanical properties: stiffness, viscoelasticity, topology, and fluid pressure, which collectively regulate cellular behavior and tissue function. Stiffness is defined as the resistance to deformation under applied force, quantified by Young's modulus. Increased ECM stiffness, driven by CAF‐mediated collagen crosslinking and deposition, promotes tumor cell invasion and mechanotransduction. The viscoelasticity is defined by the time‐dependent response to stress, wherein energy dissipation and elastic recoil are balanced. Topology refers to the structural structure defined by fibril orientation, porosity, and spatial patterns, directing cell polarity and migration. Fluid pressure refers to the hydrostatic and interstitial fluid pressures exerted by abnormal vascular and lymphatic compression, enhancing tumor cell intravascular and drug resistance.

ECM is a complex and dynamic network that plays a crucial role in various biological processes. Understanding the composition and physical properties of ECM biology is essential for unraveling its multifaceted roles in health and disease. Figure 1 illustrates the physical and mechanical characteristics of ECM, including its stiffness, viscoelasticity, and topology features, which will be thoroughly discussed in the following chapters.

### 2.1 Composition

ECM is primarily made up of collagens, which are the most abundant proteins in the human body, providing tensile strength and structural integrity [20]. Other key components include elastin, which allows tissues to resume their shape after stretching, and fibronectin, which plays a crucial role in cell adhesion and migration [21]. Additionally, elastin contributes to the resilience, stretch, and cell interactivity of the tissues [22], while glycosaminoglycans (GAGs) are essential for maintaining the structural properties of ECM and facilitating cell signaling [23]. The composition and mechanical properties of ECM show significant differences in different tissue types, anatomical regions, and pathological states. The ECM stiffness of soft tissues (like the brain) is generally low (<2 kPa) [24, 25], while hard tissues (such as bone) have a higher stiffness value (40–55 MPa) [26]. In high‐grade serous ovarian cancers (HGSOC) of the mesenchymal molecular subtype, ECM presents specific mechanical remodeling: in stiff mesenchymal tumors, stiffness was higher at the center compared with the periphery, with more than 70 kPa decrease from the center toward the edge of the tumor [27]. Breast cancer tumors are known to be stiffer (E’ = 4.04±0.9 kPa) than normal breast tissue (E’ = 0.167±0.031 kPa) [28]; during the process of pulmonary fibrosis, ECM undergoes progressive hardening, and the stiffness increase reaches 5–10 times (16.52 ± 2.25 kPa) [29].

The ECM is a highly dynamic system that constantly offers physical, biological, and chemical signals to embedded cells. Increasing evidence suggest that mechanical signals derived from the dynamic cellular microenvironment are essential controllers of cell behaviors. Physical properties of ECM such as stiffness, viscoelasticity, pore size and porosity, topology and geometry, dimensionality, and dynamic properties regulate various important biochemical and biophysical processes, such as cell adhesion, spreading, migration, growth, and differentiation [7].

### 2.2 Stiffness

In biology, stiffness usually refers to the resistance of biological tissues, cells, or other biological structures to deformation when subjected to external forces. Compelling experimental and clinical evidence has demonstrated that ECM stiffness dysregulation governs pivotal cellular processes including cell proliferation, migration, differentiation, and organoid formation [30]. In healthy tissues, the ECM exhibits a defined stiffness that contributes to tissue homeostasis. However, during disease progression, especially in cancer, ECM stiffness often increases significantly. Elevated stiffness in the tumor microenvironment (TME) has been shown to facilitate malignancy by promoting cancer cell invasiveness, enhancing immune cell infiltration, and inducing epithelial mesenchymal transition (EMT) through signaling pathways such as transforming growth factor‐beta (TGF‐β) [31, 32, 33]. For instance, stiffened ECM has been found to activate mechanotransduction pathways, such as YAP/TAZ, which regulate cell proliferation and survival. In breast cancer, higher ECM stiffness supports oncogene expression, including zinc finger protein 217 (ZNF217), and activates the protein kinase B (AKT) signaling pathway, leading to enhanced cell proliferation [34]. Similarly, in hepatocellular carcinoma (HCC), ECM stiffness (12 kPa) compared with soft ECM (1 kPa) activates the protein kinase B (PKB)/AKT, and signal transducer and activator of transcription 3 (STAT3) pathways, promoting tumor cell proliferation [35]. Furthermore, increased stiffness in pancreatic cancer tissues due to collagen crosslinking facilitates tumor growth via the tissue transglutaminase‐mediated activation of mechanosensors [36].

ECM stiffness also influences cellular mechanosensitivity. The physical properties of the matrix are sensed by integrins and other mechanosensitive receptors, which transduce mechanical signals into biochemical responses. These signals not only modulate cell adhesion and motility but also influence gene expression, such as the upregulation of ECM‐remodeling enzymes and pro‐migratory proteins. For example, in the breast tumor microenvironment, ECM stiffness has been linked to increased cell invasiveness, supporting metastasis by triggering EMT and activating pathways such as twist family BHLH transcription factor 1 (TWIST1)‐ GTPase‐activating protein SH3 domain‐binding protein 2 (G3BP2) and ephrin type‐A receptor 2 (EPHA2)/LYN proto‐oncogene, Src family tyrosine kinase (LYN)/TWIST1 [37, 38]. Additionally, stiffer ECM can enhance cancer cell aggressiveness by altering metabolic pathways and cytoskeletal dynamics, which facilitate cell migration and invasion [39]. Stiffness does more than just correlate with cancer progression; it actively enables malignant transformation through conserved force‐sensing circuits.

On the contrary, in the context of breast cancer bone metastasis, increased bone matrix mineralization, which enhances matrix stiffness, can inhibit cancer cell invasion and metastatic progression. This is achieved by disrupting the integrin‐mediated mechanosignaling pathways, including focal adhesion kinase (FAK), Rho‐associated coiled‐coil containing protein kinase (ROCK), and phosphatidylinositol 3‐kinase (PI3K), leading to reduced cell adhesion forces and migration [40]. This highlights the complexity of the mechanical properties of the ECM in modulating disease outcomes, with stiffness playing both pro‐tumorigenic and antitumorigenic roles depending on the context.

In conclusion, the stiffness of the ECM serves as a critical regulator of tumor progression. Alterations in matrix rigidity influence cancer cell behavior by promoting proliferation, invasion, and metastasis, while also modulating immune responses and ECM remodeling. The mechanical landscape of ECM is an exploitable liability in tumors. Understanding stiffness‐sensing hierarchies, from nanoscale filamin deformations to tissue‐level strain gradients, will lead to more precise mechanotherapies.

### 2.3 Viscoelasticity

Viscoelasticity refers to the dual properties of biological materials (such as cells, and tissues) that are both elastic (energy storage) and viscous (energy dissipation) when subjected to external forces. This property has important implications for cell behavior, tissue function, and disease mechanisms such as the aggressiveness of cancer cells. Recent work has revealed that the viscoelasticity of the ECM influences mechanotransduction pathways, such as mitogen‐activated protein kinase (MAPK), rat sarcoma virus oncogene (Ras), ras‐proximate‐1 (Rap1), PI3K‐AKT, Wingless‐type MMTV integration site family (Wnt), Hippo, and NF‐κB, which are involved in regulating cell behavior in response to mechanical cues [41, 42, 43, 44]. For instance, viscoelasticity has been shown to affect the behavior of HCC cells, where advanced glycation end‐products (AGEs) in the ECM enhance viscoelastic properties, promoting HCC progression through a mechanotransduction pathway involving integrin‐β1 and YAP signaling. This finding underscores the importance of viscoelasticity in cancer biology, suggesting that targeting ECM viscoelastic properties could be a novel therapeutic strategy [45].

Additionally, increased matrix viscoelasticity has been shown to accelerate and enhance the formation of vascular networks. Under hypoxic conditions, the production of reactive oxygen species (ROS) triggers the upregulation of matrix metalloproteinase‐1 (MMP‐1) and other proteases, leading to ECM degradation. This degradation results in changes to matrix viscoelasticity, which, in turn, facilitates the cluster formation of endothelial progenitor cells (EPCs) and promotes the formation of blood vessels [46].

As our understanding of matrix viscoelasticity deepens, its potential applications in both cancer therapy and regenerative medicine are becoming increasingly evident. In cancer biology, targeting the mechanistic pathways driven by ECM viscoelasticity could lead to novel therapeutic approaches aimed at disrupting tumor progression. In regenerative medicine, viscoelastic biomaterials, such as hydrogels, hold promise for improving tissue engineering by creating environments that closely mimic natural tissue properties. These materials could enable more efficient stem cell therapies and promote tissue regeneration, offering new avenues for the treatment of degenerative diseases and injuries.

### 2.4 Topology

In the field of biology, topology refers to the specific spatial arrangement of cells or the internal organization of an organism, including the relative position of cells, the nature of connections, and the shape and size of the cells. Topology involves the cellular microenvironment, including the direct physical connections between cells. Topological features directly influence various cellular processes, including adhesion, spreading, migration, and differentiation, and are crucial in shaping the cellular microenvironment, especially in the TME. By mimicking in vivo tissue architecture, the ECM provides both physical and biochemical cues that modulate cellular responses.

The 3D structure and surface topography of the ECM significantly affect cells' adherence and spread. Cells perceive and respond to the topographical features of the ECM via integrin‐mediated interactions. For example, the isotropic topological microstructure of electrospun fibers can promote the activation of integrin β1 in fibroblasts, further improve the activity of YAP signaling, and promote the phenotypic differentiation of myofibroblasts [47]. It is evident that alterations in ECM topology can exert a significant influence on cell shape and mechanical properties. This, in turn, has been shown to affect the ability of cells to interact with surrounding tissues and the extracellular environment.

The topographical features of the ECM also dictate cell migration, a process that is critical in development, wound healing, and cancer metastasis. During collective cell migration, the ECM topology plays a pivotal role in guiding cell movement. For instance, in the retinal neuroepithelial (RNE) region: the low‐porosity laminin network forms a stable mechanical anchor through high expression and activation of integrin β1, inhibits Rac1 activity, and ensures cell migration along a fixed path. The marginal region with the high‐porosity laminin network of Rac1 through local tension reduction activates Rac1 to drive cryptic lamellipodia formation and exploratory movement and finally guides cells to turn to the RNE region for directional migration [48]. Additionally, in cancer cells, the alignment of ECM fibers has been shown to affect the resistance of migrating cell clusters to chemotherapy. Aligned topography induces drug resistance through pathways like cytochrome P450 metabolism and the aryl hydrocarbon receptor (AhR), which have been linked to unfavorable prognostic outcomes in cancer [49]. This highlights the dual biomechanical and biochemical functions of ECM topography in shaping tumor development.

Topology also exerts profound effects on immune cell behavior within the TME. For example, collagen alignment in breast cancer tissues prevents the infiltration of immune cells like CD8^+^ T lymphocytes, thus contributing to immune evasion. Studies have demonstrated that inhibiting the discoidin domain receptor 1 (DDR1)‐collagen interaction can alleviate immune exclusion, presenting a promising therapeutic strategy to boost immune responses [50]. Moreover, nanogrooved surfaces mimicking aligned collagen fibers have been shown to affect T cell motility in the TME. On rigid nanogrooved surfaces, T cells exhibit mesenchymal‐like motility, whereas on soft surfaces, they adopt amoeboid‐like motility. These findings suggest that T‐cell mechanosensing is sensitive not only to stiffness but also to fiber geometry [51].

The topological features of the ECM play a crucial role in regulating cellular functions, particularly in the context of cancer. By providing topographical cues that influence cell adhesion, migration, and immune cell infiltration, ECM topology serves as a fundamental modulator of tumor progression. Understanding the molecular mechanisms by which cells perceive and respond to ECM topography will pave the way for new therapeutic strategies aimed at manipulating the TME to improve cancer treatment outcomes. Furthermore, advances in biomaterials and nanotechnology that replicate ECM topological features offer exciting opportunities to explore cellular behavior in engineered systems for both basic research and drug screening.

### 2.5 Comparison of 2D and 3D ECM Models in Vitro

More and more 2D or 3D cell cultured systems based on hydrogels or deoxy‐cell materials are being used to mimic the stiffness, topology, viscoelasticity, or other physical features of ECM. Based on the physical and chemical characteristics of ECM, the 2D culture system has revealed the key regulatory role of matrix stiffness on cell behavior. For example, by simulating different stiffness environments through polyacrylamide gel, it was found that increased stiffness can activate multiple signaling pathways to promote cell migration [52], and regulate mitochondrial dynamics and autophagy via endoplasmic reticulum‐mitochondrial calcium transport [53]. However, the 2D system cannot simulate the complexity of a real tissue microenvironment, while the 3D culture system provides a more realistic model for tumor research, drug screening, and tissue engineering by replicating tissue structure, mechanical signals, and heterogeneity [54, 55]. For example, organoid models can simulate the effect of mechanical stress in the tumor microenvironment on drug resistance [56, 57], while biodegradable scaffolds combined with growth factors can promote cartilage regeneration and maintain stem cell pluripotency [58, 59, 60].

The challenges of current 3D systems mainly focus on biocompatibility, imaging technology, and model heterogeneity reproduction. Traditional microscopic techniques make it difficult to penetrate thick 3D structures, but new technologies such as super‐resolution microscopy, light sheet microscopy, and tissue transparency methods have significantly improved deep imaging capabilities [61, 62, 63]. Advances in materials science have promoted the development of smart hydrogels and functionalized scaffolds. These materials can dynamically adjust physical characteristics in response to changes in the microenvironment and enhance accurate regulation of cell behavior [64, 65].

Several research directions are worthy of attention in the future: (1) Development of novel materials with high biocompatibility and adjustable degradation rate to accurately simulate the heterogeneity of ECM. (2) Integration of dynamic monitoring technology to track the interaction between cells and the microenvironment in real‐time through sensors. (3) Construction of personalized 3D disease models to realize accurate drug screening and individualized treatment using patient‐derived cells. With the collaborative innovation of materials science, microscopic technology, and clinical transformation, 3D culture systems will play a more important role in disease mechanism resolution, regenerative medicine, and drug development.

## 3 ECM remodeling and homeostasis

The ECM is a dynamic and complex network of proteins and carbohydrates that plays a crucial role in maintaining tissue homeostasis and regulating various biological processes. ECM remodeling is a continuous process that involves the synthesis, degradation, and modification of ECM components to maintain tissue structure and function. This process is essential for normal physiological functions such as tissue development, wound healing, and organ homeostasis [66, 67]. ECM remodeling occurs through a combination of mechanisms, including adhesion, contraction, alignment of ECM proteins, and subsequent degradation. In fibroblasts, the binding of integrin αvβ3 to fibronectin is enhanced on stiff matrices, leading to ROCK‐dependent actomyosin contraction and promoting fibrosis [68]. Myocardial fibroblasts increase the deposition of type I collagen and exacerbate cardiac fibrosis through Ras homolog gene family, member A (RhoA)/ROCK‐mediated contraction [69]. Breast cancer cells secrete lysyl oxidase‐like 2 (LOXL2), align collagen fibers to form tumor‐associated stromal channels, and accelerate metastasis [70]. In pancreatic cancer, CAFs secrete tissue inhibitors of metalloproteinase‐1 (TIMP‐1) to inhibit MMP activity, protect the dense matrix barrier, and hinder the penetration of chemotherapy drugs [71]. Cells actively participate in this process by changing the chemical, mechanical, and physical properties of their surrounding matrix. Additionally, de novo synthesis of ECM proteins, posttranslational modifications, and receptor‐mediated internalization contribute to the remodeling process [66].

The composition of the ECM varies among different organs, with each tissue having a distinct ECM profile. This specificity allows the ECM to provide tailored support and regulation for various biological structures and processes, from tissue development and elasticity to preserving the structures of entire organs [67]. For example, the brain ECM forms a perineuronal net (PNN) enriched in chondroitin sulfate proteoglycans (CSPGs), which stabilizes synaptic connections and restricts neural plasticity. Hyaluronic acid (HA) provides a hydrated matrix for glial cell migration [72]. CSPGs bind to neuronal receptors, inhibiting axon regeneration via RhoA/ROCK signaling [73, 74]. In the lung, elastin fibers allow reversible alveolar expansion, while decorin‐bound TGF‐β maintains fibroblast quiescence [75, 76, 77].

ECM remodeling is tightly regulated under normal physiological conditions. However, disruptions in this balance can lead to various pathological conditions. For instance, excessive or uncontrolled ECM remodeling is associated with fibrotic diseases and cancer progression [78]. In fibrotic diseases, such as pulmonary fibrosis, persistent TGF‐β1 signaling activates α‐smooth muscle actin positive (α‐SMA⁺) myofibroblasts, which deposit stiff collagen I. LOXL2‐mediated collagen crosslinking generates irreversible fibrils resistant to MMP cleavage. Alveolar stiffening impairs gas exchange and promotes fibroblast proliferation via YAP/TAZ mechanoactivation [79, 80, 81]. In systemic sclerosis, liver cirrhosis, and cardiovascular disease, abnormal ECM remodeling leads to excessive deposition of ECM components, resulting in tissue stiffening and impaired organ function [82, 83]. Similarly, in cancer, ECM remodeling plays a crucial role in tumor malignancy and metastatic progression [84, 85]. CAFs secrete LOXL2 and fibronectin, generating aligned collagen I fibers. These fibers act as “highways” for collective cancer cell migration [86]. ECM stiffening activates integrin β1‐FAK‐YAP signaling, which promotes the expression of prometastatic and neurotrophic genes, ultimately facilitating perineural invasion (PNI) in breast cancer [87]. Dense ECM physically blocks CD8⁺ T cell infiltration. CAF‐secreted CXCL12 recruits immunosuppressive Tregs via C‐X‐C chemokine receptor type 4 (CXCR4). CAF‐derived proline supports collagen synthesis while depleting extracellular arginine, impairing T cell function [88, 89].

MMPs and TGF‐β are two major regulators of ECM remodeling. MMPs are responsible for ECM degradation, and their activity is regulated by tissue inhibitors of metalloproteinases (TIMPs) to prevent excessive ECM breakdown [90]. In cancer, MMPs are often overexpressed, promoting ECM degradation and facilitating metastasis. For example, MMPs are highly expressed by myeloid cells in the tumor microenvironment and play a key role in degrading ECM components and enhancing tumor cell invasion [84]. TGF‐β is another critical mediator of ECM remodeling. It induces the synthesis of ECM components such as collagen and fibronectin and promotes ECM cross‐linking, leading to increased stiffness. TGF‐β also triggers EMT in tumor cells, a process that enhances their migratory capacity and facilitates invasion into surrounding tissues [91]. Additionally, TGF‐β signaling can modulate the immune microenvironment, promoting the differentiation of immunosuppressive myeloid cells that support ECM remodeling and tumor growth [92]. In summary, ECM remodeling is a crucial process that regulates tissue homeostasis and contributes to the pathogenesis of various diseases. Understanding the mechanisms of ECM remodeling and its impact on disease will provide valuable insights for the development of therapeutic strategies aimed at targeting ECM remodeling in cancer, fibrosis, and other diseases.

## 4 ECM Mechanotransduction Mechanisms

**FIGURE 2:**
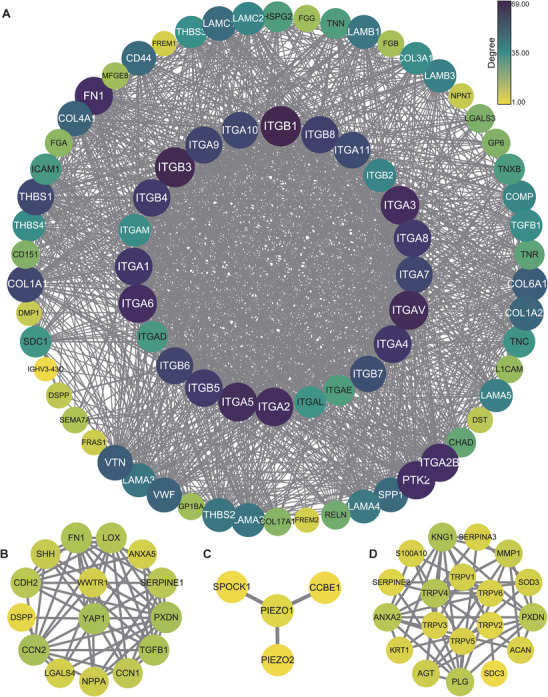
Protein–protein interaction (PPI) networks of ECM proteins corresponding to mechanotransduction proteins. (A) PPI network related to integrin. (B) PPI network related to YAP/TAZ. (C) PPI network related to PIEZO. (D) PPI network related to TRPV. Each circle in the figure represents a protein, labeled with its corresponding gene name. The color of the circles corresponds to the degree scale on the right, indicating the connectivity of each protein within the network. Darker colors denote proteins with a higher number of interactions, suggesting greater potential significance in the network. The lines between circles represent protein‐protein interactions. ITGA, Integrin α; ITGB, Integrin β; PIEZO1, Piezo1; PIEZO2, Piezo2; TRPV, TRPV; YAP, YAP1; TAZ, WWTR1. GO: 0031012.

Mechanical signals from the ECM are detected and transduced by specialized receptors, primarily integrins, which mediate the bidirectional communication between the ECM and the cytoskeleton. This process, known as mechanotransduction, allows cells to sense and respond to changes in the physical properties of the ECM, including stiffness, topography, and elasticity. We performed the protein‐protein interaction networks functional enrichment analysis by an online data resource (STRING: functional protein association networks: https://cn.string‐db.org), it exhibits the complex interactions between mechanosensors and matrix proteins, suggesting the complexity and diversity of mechanotransduction (Figure 2).

### 4.1 Integrin Mediated Mechanotransduction

Integrins are heterodimeric transmembrane receptors composed of alpha and beta subunits, particularly αvβ6, αvβ3, and α5β1, which function as mechanosensors that respond to ECM stiffness [93, 94, 95]. Integrins respond to mechanical cues from the ECM through both “inside‐out” and “outside‐in” signaling mechanisms. In “outside‐in” signaling, ECM binding induces integrin clustering, which activates intracellular signaling pathways. Conversely, “inside‐out” activation involves cell‐generated signals that alter integrin affinity and promote ECM interactions [96, 97, 98]. Stiffness or topography directly influences integrin‐mediated mechanotransduction. For instance, increased ECM stiffness enhances integrin signaling, leading to cytoskeletal reorganization, cellular migration, and ECM remodeling. This process is essential in various physiological and pathological contexts, such as tissue development, fibrosis, and cancer progression. In fibrosis, matrix crosslinking, which occurs through integrin‐mediated activation of enzymes like lysyl oxidase (LOX), results in ECM stiffening, promoting pathological remodeling and exacerbating disease [99, 100].

Moreover, integrins engage in reciprocal interactions with other mechanosensitive receptors, such as syndecans and discoidin domain receptors (DDRs), to amplify ECM signals. Syndecans link ECM components to integrins and activate downstream signaling cascades involving focal adhesion kinase (FAK) and RhoA, thereby promoting cell spreading and ECM remodeling [96]. Mechanoreceptors also interact with matrix proteins. Changes in stiffness can alter intracellular mechanical signal transduction through aggregation and activation of integrins. Adhesion of integrins to the extracellular matrix triggers downstream FAK activation, which then recruits Src to bind to integrins, which in turn enhances FAK activity [101]. The activation of integrins further triggers the activation of intracellular signaling pathways through focal adhesion kinase phosphorylation (pY397‐FAK), thereby regulating cell behavior and fate (such as proliferation, migration, and differentiation) [102]. Additionally, integrin‐mediated signaling contributes to the development of tumors by modulating cell proliferation, survival, and invasion through pathways such as FAK, MAPK and RhoA/ ROCK signaling [94, 103, 104, 105, 106]. Integrin‐linked kinases (ILKs) and paxillin have also been reported to be important junctions for integrin‐mediated fibrosis. Actin is linked to myosin II and transmits biomechanical signals to the nucleus. In this process, YAP and TAZ with PDZ‐binding motifs are translocated into the nucleus to facilitate downstream gene transcription in cell proliferation, collagen synthesis, and cell differentiation. This, in turn, increases ECM stiffness [95]. At the same time, mechanical signals are passed between integrin molecules, activating PI3K/AKT signaling and the connexin hemichannel to mediate mechanical transduction [107]. The modulation of FAK activity by mechanical forces, such as tension and shear stress, is essential for cellular responses to changes in the ECM, particularly in the context of tissue remodeling and cancer metastasis [108, 109].

As integral components of ECM mechanotransduction, integrins not only sense mechanical changes but also drive cellular responses that influence disease outcomes. Given the therapeutic potential of integrin‐targeted therapies, such as inhibitors of α4β7, αvβ6, and α5β1 [110, 111, 112], further research into the specific roles of integrins and their interaction with other cellular signaling pathways is crucial for developing more effective, tissue‐specific interventions. Integrin‐mediated mechanotransduction represents a key mechanism by which cells interpret and respond to the biophysical properties of their environment, with profound implications for tissue remodeling, disease progression, and therapeutic strategies.

### 4.2 Ion Channels (Piezo1 and TRPV4) Mediated Mechanotransduction

Ion channels are a special class of transmembrane proteins that can regulate their conformational changes through physical signals (such as mechanical stress, temperature, pressure, or light) to mediate the transmembrane flow of ions inside and outside the cell, thereby dynamically regulating cell functions. Piezo1 and TRPV4 represent the most significant ion channels. Piezo1 is a constituent of the piezo family of mechanically activated cation channels [113] and TRPV4 belongs to the TRP superfamily of nonselective cation channels [114]. These channels integrate mechanical information encoded in ECM stiffness and topography into downstream biochemical pathways, thereby shaping tissue homeostasis and pathological remodeling.

Piezo1, a large trimeric cation channel, exhibits exquisite sensitivity to membrane tension and substrate rigidity. Upon mechanical stimulation, Piezo1 rapidly transitions into an open state, allowing calcium influx that triggers signaling cascades including Ca^2^⁺/calmodulin‐dependent protein kinase II (CaMKII) [115, 116], endothelial nitric oxide synthase (eNOS) [117], and small GTPases such as RhoA [118, 119]. In endothelial cells, Piezo1 activation under shear stress is critical for vascular tone regulation and blood flow adaptation [120]. Notably, in fibrotic contexts, Piezo1 functions as a mechanosensor of matrix stiffening. Elevated ECM stiffness upregulates Piezo1 expression, which in turn amplifies fibroblast activation through a Wnt2/11–C‐C motif chemokine ligand 24 (CCL24) axis, establishing a feedforward loop that exacerbates matrix deposition and tissue rigidity. Genetic or pharmacological inhibition of Piezo1 disrupts this circuit and mitigates fibrosis, suggesting a promising therapeutic avenue for stiffness‐driven diseases [121]. In addition to stiffness, ECM topography modulates Piezo1 activation, though the precise structural determinants, such as nano‐ridge spacing and curvature sensitivity—remain incompletely resolved and warrant further high‐resolution structural and biophysical investigation [122].

Functionally intertwined with Piezo1, TRPV4 is a nonselective calcium‐permeable cation channel responsive to osmotic, thermal, and mechanical stimuli. Although TRPV4 lacks intrinsic mechanosensitivity, its activation is facilitated by mechanotransducing partners such as integrins and cytoskeletal linkers. In load‐bearing tissues like cartilage, TRPV4 mediates anabolic signaling under physiological strain, promoting collagen II and aggrecan expression while suppressing catabolic enzymes like MMPs [123]. This Ca^2^⁺‐dependent activity preserves ECM integrity and confers resistance to mechanical degeneration. However, in pathological settings, TRPV4 is co‐opted by stiff ECM microenvironments to induce EMT and fibrotic progression via AKT–YAP signaling [124, 125]. Intriguingly, elevated mechanical stress in pancreatic fibrosis has been shown to initiate a cooperative Piezo1–TRPV4 axis, wherein Piezo1‐mediated calcium entry enhances TRPV4 conductance, driving stellate cell activation and chronic tissue damage [126].

Emerging evidence suggests both Piezo1 and TRPV4 are not confined to structural or stromal cells but also modulate immune cell function within mechanically dynamic niches such as tumors. For instance, Piezo1 co‐localizes with TLR4 in macrophages, orchestrating reactive oxygen species (ROS) production and enhancing pathogen clearance through a CAMKII–MST1/2–Rac pathway [115]. TRPV4 has similarly been implicated in regulating immune cell migration and polarization [127, 128], yet its precise contribution to antitumor immunity and immune–ECM crosstalk remains poorly characterized. The potential of TRPV4 to act as a mechano‐immunomodulator, especially in the context of tumor stiffness and immune exclusion, represents an important but underexplored frontier.

Despite significant advances, several open questions persist. The molecular basis by which ECM topography and roughness regulate Piezo1 gating remains elusive, likely requiring integrative Cryo‐EM, membrane reconstitution, and atomic force microscopy approaches. Furthermore, the cooperative or antagonistic interactions between Piezo1, TRPV4, and other mechanosensitive pathways (e.g., integrin–focal adhesion signaling, Hippo–YAP/TAZ axis) require clarification to delineate context‐specific channel function. Finally, therapeutic targeting of mechanosensitive ion channels must account for their pleiotropic roles in physiology and pathology, underscoring the need for cell‐type‐specific modulators that can disrupt pathological signaling while preserving essential mechanosensing functions.

Together, Piezo1 and TRPV4 serve as crucial molecular relays in the translation of ECM mechanical properties into intracellular signals, orchestrating responses across a wide spectrum of cell types and tissues. Their roles in integrating mechanical, inflammatory, and fibrotic cues underscore their potential as master regulators of mechano‐adaptive and mechano‐pathological responses, and as promising targets in the design of precision therapies for fibrosis, cancer, and immune dysfunction.

### 4.3 YAP/TAZ Mediated Mechanotransduction

YAP and TAZ, homologous proteins that function as transcriptional co‐activators, YAP and TAZ are key effectors of the Hippo signaling pathway, which regulates organ size, tissue homeostasis, and fibrosis. The activation of YAP/TAZ is strongly influenced by mechanical cues from the ECM, such as stiffness, cell geometry, and mechanical stress. When the ECM becomes stiffer, particularly in pathological conditions like fibrosis or cancer, the mechanical forces trigger the activation of YAP/TAZ through various signaling pathways, including the Hippo pathway and non‐Hippo dependent mechanisms. In normal physiological conditions, YAP/TAZ remain cytoplasmic and are sequestered by phosphorylation and degradation processes. However, in response to mechanical signals, unphosphorylated YAP/TAZ translocate to the nucleus, where they interact with DNA‐binding partners, notably TEA domain transcription factors (TEADs), to regulate the expression of genes involved in cell proliferation, survival, and ECM remodeling [129]. It is evident that the primary upstream inhibitor of Hippo pathway‐mediated activation of YAP/TAZ is the Hippo (MST1/2‐LATS1/2) pathway, while the key upstream activators are mechanically induced integrin‐SRC and E‐cadherin‐ Ajuba LIM protein (AJUBA)/thyroid receptor interacting protein 6 (TRIP6)/IM domain containing 1 (LIMD1). Upstream activators and inhibitors regulating YAP and TAZ have been reviewed elsewhere [130].

Mechanistically, ECM stiffness enhances integrin clustering and focal adhesion formation, activating the integrin‐FAK‐RhoA‐ROCK axis, which drives cytoskeletal tension and F‐actin polymerization. This reorganization of the actin cytoskeleton increases nuclear stiffness via laminA dephosphorylation and cytoskeletal coupling to the nucleus through the LINC complex, facilitating nuclear pore dilation and YAP/TAZ nuclear entry. Once in the nucleus YAP/TAZ associate with transcription factors, particularly TEADs, to orchestrate gene expression programs involved in proliferation, survival, and ECM remodeling [125, 129]. Recent evidence further suggests cooperation with activator protein 1 (AP‐1), myocardin‐related transcription factor (MRTF), and serum response factor (SRF), reinforcing their role in driving fibroblast activation and tumor progression [130].

Pathological matrix stiffening acts as a sustained cue for YAP/TAZ activation in various diseases. In cardiac fibrosis, for example, elevated ECM stiffness activates Angiotensin II receptor type 1 (AT1R)‐mediated YAP/TAZ signaling, promoting myofibroblast differentiation and excess matrix deposition [131]. Similarly, in tumor microenvironments, YAP/TAZ activation is enhanced by mechanical tension and facilitates EMT, metastasis, and cancer stemness [132, 133, 134]. In prostate cancer models, substrates of pathological stiffness (∼46.7 kPa) have been shown to promote nuclear accumulation of YAP/TAZ, correlating with increased metastatic potential [135]. Additionally, TGF‐β and Wnt pathways converge on YAP/TAZ activation in fibroblasts, while crosstalk with the Smad2/3 axis reinforces fibrogenic transcription [136, 137].

A comprehensive view of this mechanotransduction network reveals a three‐step model: (1) Stiffness signal conduction: Integrin‐FAK‐RhoA pathway. Matrix stiffness enhances integrin clustering → activates FAK → RhoA‐GTP membrane localization → drives ROCK (myosin contraction) and mDia (F‐actin polymerization). (2) Cytoskeleton reorganization: The concentration of F‐actin determines the rigidity of the cytoplasm, affects the dephosphorylation of lamin A, and enhances the rigidity of the nuclear membrane, stabilizing the nuclear localization of YAP/TAZ. (3) Nuclear mechanical coupling: Stress fibers stretch the nuclear membrane through the LINC complex → expand the diameter of nuclear pores → increase the rate of YAP/TAZ nuclear input [138]. This integrative mechanism underscores how cells decode mechanical signals to regulate their fate and function.

From a translational standpoint, targeting YAP/TAZ mechanosignaling offers a promising therapeutic strategy in diseases characterized by aberrant ECM stiffening. In cancer, disrupting the YAP/TAZ mechanotransduction loop may attenuate invasive behavior and chemoresistance. In fibrotic and glaucomatous tissues, pharmacological modulation of upstream activators, including Rho‐associated kinases and mechanosensitive channels, could prevent pathological matrix remodeling. Furthermore, viscoelastic biomaterials engineered to modulate YAP/TAZ activity represent an emerging frontier in regenerative medicine. As our understanding of ECM–YAP/TAZ interactions deepens, new opportunities arise for precise mechanical control of cell function in both pathological and therapeutic contexts.

**FIGURE 3:**
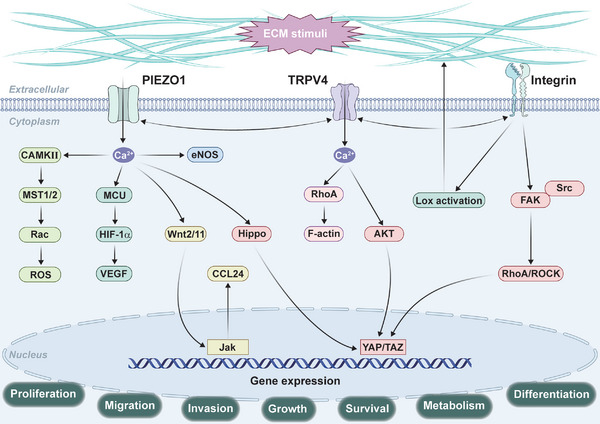
Schematic representation of mechanotransduction pathways. Cell integrates mechanical cues (matrix stiffness, viscoelasticity, topology, and fluid pressure) from their microenvironment to regulate inflammatory response, wound healing, angiogenesis, and migration.

ECM mechanotransduction is a fundamental process that regulates cellular behavior in response to mechanical cues such as ECM stiffness, shear stress, and traction force. Key signaling pathways such as integrin, Piezo1, TRPV4, and YAP/TAZ play central roles in translating these mechanical signals into cellular responses (Figure 3). Understanding the dynamic mechanisms by which cells interact with the ECM and adapt to mechanical changes is crucial for developing therapeutic strategies for diseases such as fibrosis, cancer, and cardiovascular disorders. The regulation of ECM remodeling through mechanotransduction offers promising avenues for intervention in these pathologies.

## 5 ECM in Physiological Functions

The ECM is not merely a passive scaffold; rather, it actively influences cellular behavior through biochemical and mechanical signals. For instance, the stiffness of the ECM can determine cell fate, such as differentiation and migration, by modulating intracellular signaling pathways [139]. Stiffening prompted the intestinal stem cells to preferentially differentiate toward goblet cells. Moderate substrate stiffness promoted breast cancer cell motility. In addition, matrix stiffness regulates mitochondrial fusion and fission in breast cancer cells, directly affecting apoptosis and autophagy in breast cancer cells [140, 141, 142]. These mechanobiological principles extend beyond individual cell fate decisions to orchestrate systemic physiological responses, particularly in immune regulation and tissue repair.

### 5.1 The Role of ECM in Immune Response

The ECM plays a pivotal role in regulating immune responses by influencing the polarization, activation, and migration of immune cells. The mechanical properties of ECM can significantly alter immune cell behavior within the TME and other tissue settings. In particular, ECM stiffness is a key factor that modulates the functional state of immune cells, particularly macrophages and T cells, through direct interactions with ECM components. ECM stiffness is often increased in pathological conditions like cancer, fibrosis, and inflammation, and can impede immune cell infiltration by creating a physical barrier. For example, in tumors, dense ECM components, particularly collagen crosslinks, restrict T‐cell migration into tumor islets, preventing effective immune surveillance and response [143]. Collagen fibers, when heavily crosslinked and oriented in parallel arrays, form a rigid, fibrotic structure that hinders the movement of immune cells such as T cells, thereby contributing to immune evasion by the tumor [144]. Notably, the matrix stiffness increases the CD4/CD8 T cell ratio, but simultaneously suppresses the activation of CD8^+^ T cells, a hallmark of immune dysfunction in solid tumors [145].

Mechanistically, ECM‐induced mechanical signaling, through integrin receptors and mechanosensitive pathways like RhoA/ROCK and YAP/TAZ, leads to alterations in the cytoskeletal dynamics of T cells, decreasing their activation and proliferation [146]. Recent evidence highlights biomechanical stress also drives CD8^+^ T cell exhaustion in solid tumors via a signaling cascade centered on the transcription factor odd‐skipped related transcription factor 2 (Osr2). Osr2 recruits histone deacetylase 3 (HDAC3) to reprogram the epigenetic landscape of exhausted CD8^+^ T cells, suppressing cytotoxic gene expression and enhancing T cell exhaustion. This reveals Osr2 as a biomechanical checkpoint that exacerbates T‐cell exhaustion, offering a potential target to enhance cancer immunotherapy [147].

Beyond T cells, ECM stiffness can affect macrophage polarization, with soft matrices promoting proinflammatory M1 polarization and stiffer matrices favoring anti‐inflammatory M2 polarization [84, 148]. Importantly, the interaction between the ECM and immune cells is bidirectional. Immune cells actively remodel ECM composition, while ECM components influence immune cell activation through receptor–ligand interactions. For example, HA, through its interaction with receptors such as CD44, can promote T‐cell recruitment to inflamed tissues [149]. In parallel, collagen can bind to the inhibitory receptor leukocyte‐associated immunoglobulin‐like receptor‐1 (LAIR‐1) on T cells, leading to immune suppression in the TME [150]. Additionally, ECM‐bound molecules like collagen triple helix repeat containing 1 (CTHRC1) and transforming growth factor‐beta‐induced protein (TGFBI), secreted by fibroblasts, can modulate macrophage activity and cytokine production, which in turn affects tumor immune responses [151]. This dynamic crosstalk establishes a feedback loop in which ECM remodeling and immune function continuously influence each other.

Together, these findings underscore a finely tuned mechanobiological dialogue between the ECM and the immune system. Dissecting the spatial and mechanical cues within the ECM microenvironment offers a promising avenue to modulate immune cell behavior, overcome immune evasion, and enhance the efficacy of immunotherapies. Future studies should aim to unravel the spatiotemporal dynamics of ECM stiffness sensing and to develop biomaterials or pharmacological tools that can reprogram the mechanical–immune axis for therapeutic benefit.

### 5.2 ECM in Angiogenesis, Tissue Homeostasis and Repair

In angiogenesis, ECM acts as a scaffold, guiding cell behavior and facilitating the regenerative process. Angiogenesis, the formation of new blood vessels from pre‐existing ones, is a critical step for tissue repair, supplying necessary nutrients and oxygen to the regenerating tissues. ECM components, including fibronectin, collagen, and glycosaminoglycans, are essential for angiogenesis. They not only provide a structural scaffold for the formation of new vessels but also interact with specific receptors on endothelial cells to modulate angiogenic signaling. For example, integrins such as β3 and β5 are involved in the activation of endothelial cells through pathways like FAK/extracellular signal‐regulated kinase (ERK), promoting endothelial cell migration and vessel formation [152, 153, 154]. Additionally, the mechanical properties of ECM, such as stiffness and topography, modulate these processes by altering integrin‐mediated signaling and affecting vascular integrity. ECM remodeling, driven by MMPs, supports the breakdown of the basement membrane and facilitates endothelial cell movement during vessel formation [155, 156].

In tissue repair, ECM components such as collagen and fibronectin are crucial for the deposition of a provisional matrix that supports cell migration and proliferation. The remodeling of this matrix is essential for restoring tissue architecture and function. M2 macrophages play a significant role in ECM remodeling during the wound‐healing process. They secrete ECM proteins like collagens and fibronectin, promoting tissue regeneration, while also regulating ECM turnover through enzymes like MMPs and their inhibitors, TIMPs. These macrophages balance ECM degradation and deposition, ensuring proper tissue regeneration and preventing fibrosis [157, 158, 159, 160, 161, 162, 163].

The composition, mechanical properties, and remodeling dynamics of the ECM determine whether repair culminates in functional tissue regeneration or maladaptive fibrosis. Future therapeutic strategies may benefit from targeting ECM stiffness and composition, as well as modulating macrophage phenotypes, to promote tissue regeneration while avoiding maladaptive remodeling. Emerging biomaterials that mimic the dynamic and mechanical properties of native ECM offer exciting avenues for guiding vascularization and tissue repair in engineered systems and regenerative medicine.

## 6 ECM Dysregulation in Diseases

**FIGURE 4:**
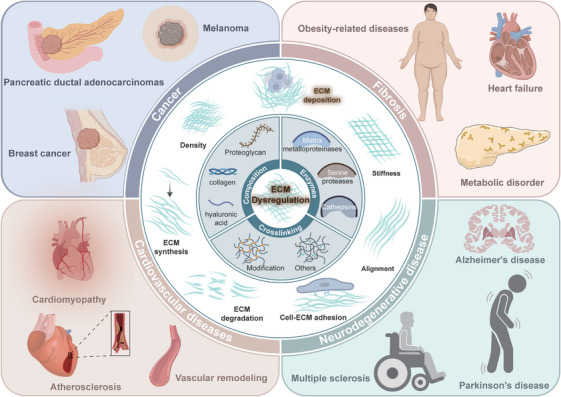
ECM‐related diseases. The main components and physical characteristics of ECM engage in several diseases. These include cancers, such as breast cancer and melanoma and metabolic diseases, including obesity‐related diseases, heart failure, and metabolic disorders. Additionally, it has been associated with cardiovascular diseases, such as cardiomyopathy and atherosclerosis, as well as neurodegenerative diseases, including Alzheimer's disease and Parkinson's disease.

Dysregulation of the ECM is implicated in a wide range of diseases, including cancer, fibrosis, and autoimmune disorders. The role of ECM in disease is multifaceted, involving changes in its composition, structure, and interaction with cells, which can lead to altered cell behavior and disease progression (Figure 4).

### 6.1 Cancer

In cancer, the ECM is a critical component of the tumor microenvironment, influencing tumor growth, invasion, and metastasis. The ECM in tumors often undergoes significant remodeling, characterized by increased stiffness and altered composition, which can promote malignant transformation and resistance to therapy. For instance, the interplay between cancer cells and the ECM in the tumor microenvironment is crucial for understanding tumorigenesis and identifying therapeutic targets [164]. Moreover, in melanoma and breast cancer, increased amounts of collagen I and collagen III correlated with disease progression and reduced survival [165, 166, 167, 168]. In pancreatic ductal adenocarcinoma (PDAC), a disruption of the balance between ECM synthesis and secretion and the altered expression of matrix remodeling enzymes lead to abnormal ECM dynamics in PDAC. This pathological ECM promotes cancer growth, survival, and invasion and alters the behavior of fibroblasts and immune cells leading to metastasis formation and chemotherapy resistance, which contribute to the high lethality of PDAC [169]. The study demonstrated that bone morphogenetic protein1 (BMP1) has the capacity to impede tumor growth and metastasis in PDAC models by fostering fibrillar collagen deposition [170]. However, this effect was observed exclusively in cancer cells that exhibited substantial collagen production. A compelling question for future research is whether the distinct functions of BMP1 in other cancers are associated with the levels of collagen produced. In the event of PDAC being treated with an ECM depletion agent, such as an FAK inhibitor, there is the potential for the abnormal expression of STAT3 to hinder the function of the FAK inhibitor. This, in turn, can result in the development of drug resistance [171]. Furthermore, COL11A1/AKT/cyclic AMP response‐element binding protein (CREB) in PDAC sustains cell survival against detachment‐induced anoikis [172]. The interplay between cancer cells, immune cells, and the ECM within the TME has been demonstrated to contribute further to disease progression [173].

### 6.2 Fibrosis

ECM dysregulation plays a pivotal role in the development of fibrosis across a range of organs. ECM remodeling leads to fibrosis, causing stiffness and heart failure [174]. Adipose tissue fibrosis, characterized by ECM overaccumulation, contributes to obesity‐related metabolic disorders [175]. Key molecular mechanisms underlying fibrosis include angiotensin II, transforming growth factor‐β1, inflammation, and oxidative stress [176]. These factors activate signaling pathways like Angiotensin II (Ang‐II)‐MAPK and TGF‐β1‐Smad, inducing the expression of profibrotic genes. ECM crosslinking modifications, regulated by specific enzymes, alter ECM structure and function, contributing to fibrosis progression. The balance between MMPs and their inhibitors is critical in ECM metabolism and fibrosis [176, 177]. Understanding these mechanisms is essential for developing targeted therapies to combat fibrosis and related diseases across different tissues.

### 6.3 Cardiovascular Diseases

In cardiovascular tissues, the ECM provides structural support and regulates the biomechanical properties essential for normal heart and vascular function. Alterations in ECM composition and organization are closely associated with the development and progression of various cardiovascular conditions, including cardiomyopathy, atherosclerosis, and vascular remodeling. In hypertrophic cardiomyopathy (HCM), sarcomere dysfunction leads to reduced cardiomyocyte secretion of ECM ligands and altered fibroblast interactions, promoting nonmyocyte phenotypes [178]. Early‐stage HCM is characterized by dysregulated ECM remodeling, altered integrin expression, and disrupted cell‐ECM adhesion [179]. Cardiac fibrosis, a common feature of cardiomyopathies, results from excessive ECM protein deposition and fibroblast activation, leading to tissue stiffness and heart failure [174].

In atherosclerosis development and progression, ECM remodeling affects plaque stability, cellular migration, and inflammatory responses. This dynamic remodeling is driven by proteolytic enzymes such as MMPs, cathepsins, and serine proteases [180]. Recent research highlights the importance of proteoglycans–glycosaminoglycans synthesis and remodeling, as well as elastin metabolism, in disease progression [181]. Noncoding RNAs have emerged as significant regulators of ECM remodeling and plaque progression [182]. For example, the overexpression of miR‐181a‐5p and miR‐181a‐3p in ApoE mice decreased the plaque size [183]. Understanding ECM dynamics offers the potential for developing novel therapeutic strategies and diagnostic tools for atherosclerosis and related cardiovascular diseases [180, 181].

### 6.4 Extracellular Matrix Alterations in Neurodegenerative Disease

In neurodegenerative diseases, such as Alzheimer's disease (AD), Parkinson's disease, and multiple sclerosis, ECM components undergo significant remodeling, which can exacerbate disease pathology. In Alzheimer's disease, ECM remodeling is linked to amyloid‐β deposition. ECM remodeling can activate astrocytes and enhance the autophagy‐lysosome pathway, aiding in amyloid‐beta clearance and alleviating Alzheimer's pathology [184]. Furthermore, studies have revealed spatially segregated molecular rearrangements of ECM components in AD brains, including reduced brevican and increased neurocan, aggrecan, hyaluronan, and proteoglycan link protein 1 (HAPLN1) levels [185].

Parkinson's disease also exhibits ECM alterations, particularly in the striatal region. Oxidative stress‐induced chemical modification of ECM, such as protein carbonylation, oxidation of lipids and carbohydrates to form cross‐linked structures, increases the stiffness of ECM, changes the chemical composition of ECM, affects the survival and morphology of microglia, reduces the tension of the cell skeleton, activates mechanoreceptors, exacerbates neuroinflammation, limits the effect of cell replacement therapy, and ultimately accelerates the progression of Parkinson's disease [186]. Additionally, ECM components like hyaluronan can act as diffusional barriers, impacting the propagation of pathological proteins such as alpha‐synuclein [187].

In multiple sclerosis, ECM molecules deposited into lesions create an inhibitory microenvironment that impedes remyelination. The altered ECM can prevent the recruitment and differentiation of oligodendrocyte progenitor cells, hindering the repair process. Moreover, ECM components can exacerbate inflammatory responses, further contributing to disease progression [188]. The role of ECM in neurodegenerative diseases extends beyond structural support. Extracellular vehicles (EVs), which carry ECM components, have been implicated in the pathogenesis of these diseases. EVs can serve as biomarkers and potential therapeutic targets, offering insights into disease mechanisms and progression [189, 190].

Overall, understanding ECM alterations in neurodegenerative diseases is crucial for developing therapeutic strategies. Targeting ECM components and their interactions with cellular processes may provide new avenues for treatment, potentially slowing disease progression and improving patient outcomes [191, 192].

## 7 Therapeutic Strategies

**FIGURE 5:**
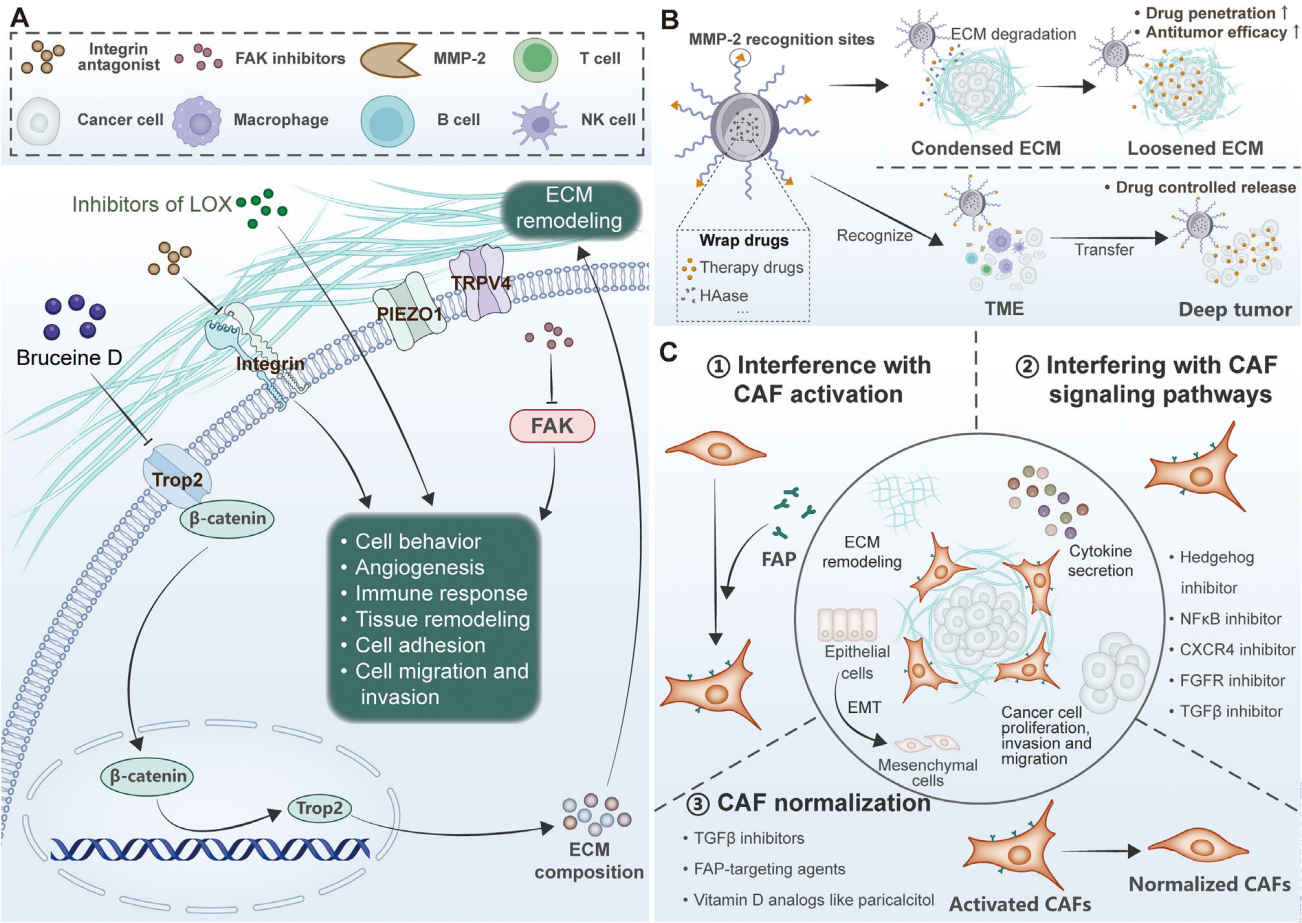
ECM targeting therapeutic strategies. Various players in ECM mechanotransduction have been proposed as attractive therapeutic targets, including CAFs, Pioze1, Trop2, and ECM remodelers. The above treatment methods regulate drug penetration, TME activation, ECM remodeling, antiangiogenesis, and the activation and modulation of CAFs. Specifically, hyaluronidase (HAase) and drugs alter ECM properties, enhancing drug diffusion and triggering TME responses. MMPs regulate ECM degradation and control drug release. The mechanisms of antiangiogenesis is a tumor growth suppression strategy, and the signaling pathways involved in CAFs activation and normalization play an essential role. These elements collectively provide insights into tumor progression and potential therapeutic interventions targeting ECM dynamics, CAFs modulation, and tumor vascularization.

Targeting ECM components, such as integrins and matrix metalloproteinases, as well as ECM‐remodeling cells, has shown potential as a therapeutic strategy, although with limited success so far [96]. ECM crosslinking, governed by specific enzymes, contributes to fibrosis across various organs. Targeting these crosslinking pathways is being explored as a therapeutic approach to restore normal tissue structure and function in fibrotic diseases [177]. Understanding ECM dysregulation in these diseases provides insights into potential diagnostic biomarkers and therapeutic targets for future research and treatment development. Figure 5 describes ECM‐targeted strategies use mechanotransduction components to modulate drug delivery, tumor microenvironment activation, and matrix reorganization.

### 7.1 Small Molecule Therapeutic Drugs Targeting ECM

Targeting the ECM with small molecule inhibitors represents a promising strategy for therapeutic intervention in various pathologies, including cancer, fibrosis, and neurodegenerative diseases. By modulating ECM turnover and the mechanical properties of the tumor microenvironment, small molecule drugs can alter tumor cell behavior, angiogenesis, immune response, and tissue remodeling. In cancer, ECM remodeling facilitates tumor progression and metastasis through the synthesis, deposition, and degradation of ECM proteins, which influence tumor cell adhesion, migration, and invasion. For instance, PEGPH20, a pegylated recombinant human hyaluronidase, targets HA, a key ECM component often overproduced in solid tumors. By degrading HA, PEGPH20 reduces tumor interstitial pressure, enhances chemotherapy drug delivery, and reprograms the ECM to a more permissive state for therapeutic intervention. Despite promising preclinical data, clinical trials have yielded mixed results, highlighting the complex nature of ECM‐targeted therapies. Notably, combination strategies using PEGPH20 with immune checkpoint inhibitors, such as pembrolizumab, have shown improved outcomes in certain subsets of patients with high HA expression (NCT02563548) [193].

Furthermore, Cilengitide, an integrin antagonist targeting the αvβ3 and αvβ5 integrins, disrupts the interaction between tumor cells and the ECM, thereby inhibiting cell adhesion, migration, and angiogenesis. This therapeutic strategy has demonstrated efficacy in various solid tumors, including glioblastoma and non‐small‐cell lung cancer (NSCLC), and has entered late‐phase clinical trials (NCT01118676). However, Integrin inhibition has faced challenges related to tumor heterogeneity and pharmacokinetic limitations, underscoring the need for more precise targeting strategies [194].

In addition to integrins, small molecule inhibitors also target ECM‐modifying enzymes, such as LOX, which catalyzes the cross‐linking of collagen and elastin, thereby increasing ECM stiffness—a critical factor in tumor metastasis and fibrosis. Inhibitors of LOX, such as PXS‐5505, have demonstrated preclinical efficacy in reducing ECM stiffness and inhibiting tumor progression, particularly in pancreatic cancer. Simtuzumab, a monoclonal antibody targeting LOXL2, was tested in phase II trials for pancreatic cancer and idiopathic pulmonary fibrosis, but clinical efficacy has been limited (NCT01672853) [195, 196, 197].

Another promising approach involves targeting FAK, a key regulator of cell‐ECM interactions, which has emerged as a novel therapeutic approach. FAK inhibitors, such as GSK2256098 and PF‐00562271, have been evaluated in phase I and II clinical trials, demonstrating the potential to modulate ECM remodeling and tumor progression. These inhibitors disrupt mechanotransduction pathways that mediate cell migration, proliferation, and survival, which are often dysregulated in cancer and fibrosis [198, 199, 200, 201]. However, FAK inhibition has shown limited success in clinical trials, necessitating further optimization and combination with other therapies (NCT02551653). Additionally, LOX induces ferroptosis in nerve cells in a GPX4‐dependent manner by catalyzing collagen I/III cross‐linking to increase ECM stiffness and activate the mechanosensitive channel Piezo1. Small molecule LOX inhibitors (such as traumatic acid TA) or piezo1 antagonists can reverse ECM sclerosis and improve cognitive dysfunction after brain injury, highlighting the neuroprotective value of the regulation of matrix mechanical properties [202]. Bruceine D (BD) targets the Lys307/Glu310 checkpoint of trophoblast cell surface antigen 2 (Trop2), disrupts the formation of the Trop2/β‐catenin complex, and blocks the nuclear translocation of β‐catenin, thereby inhibiting Trop2 transcription, EMT progression, and ECM remodeling [203]. This mechanism reveals the dual role of small molecule drugs targeting ECM‐related membrane proteins in antimetastatic therapy: both regulating signaling pathways and indirectly remodeling the stromal microenvironment [203].

Finally, ion channel modulators such as GSK1016790A (TRPV4 agonist) and Dooku1 (Piezo1 inhibitor) have shown promise in preclinical models [204, 205]. However, translating these findings into clinical applications requires precise targeting strategies to minimize off‐target effects and adverse mechanical signaling disruptions. Small‐molecule therapeutic drugs targeting the ECM offer a promising avenue for cancer and fibrosis therapy, particularly when used in combination with traditional chemotherapies or immunotherapies. Despite the therapeutic potential, challenges remain in terms of efficacy, tumor heterogeneity, and off‐target effects. Future research will likely focus on developing more specific inhibitors, identifying predictive biomarkers, and optimizing combination therapies to enhance therapeutic outcomes and overcome current limitations. Currently, ongoing clinical drug trials targeting ECM are partially summarized in Table 1.

**TABLE 1:** Summary of clinical research on ECM‐targeting drugs.

Agent	Disease Area	Therapeutic Approach	Target	Mechanism	Clinical Stage	NCT Number	Refs
PEGPH20	Various solid tumors	ECM degradation enzyme therapy	HA	Degrades HA to reduce tumor interstitial pressure and enhance chemotherapy penetration.	Phase II	NCT02715804	[193, 206]
Cilengitide	Glioblastoma NSCLC	Integrin blockade	αvβ3/αvβ5	Inhibits tumor cell‐ECM adhesion and angiogenesis.	Phase III/ Phase I	NCT00689221/ NCT01118676	[194, 207]
Etaracizumab (MEDI‐522)	Metastatic melanoma	Monoclonal antibody therapy	αvβ3	Blocks integrin‐mediated metastasis signaling.	Phase II	NCT00066196	[208]
GSK2256098	Pancreatic cancer Pulmonary hypertension	Kinase inhibition	FAK	Inhibits FAK‐mediated mechanotransduction.	Phase I	NCT02551653	[198]
PF‐00562271	Pancreatic cancer Head and neck neoplasms	FAK inhibition	FAK	Inhibits FAK, modulating ECM remodeling and tumor progression.	Phase I	NCT00666926	[209]
Defactinib	Malignant pleural mesothelioma (MPM)	Dual‐target inhibition	FAK/PTK2	Disrupts tumor cell migration and microenvironment remodeling.	Phase II	NCT01870609	[210]
Simtuzumab	Primary sclerosing cholangitis (PSC)	Monoclonal antibody therapy	LOXL2	Inhibits collagen crosslinking and reduces tissue stiffness.	Phase II	NCT01672853	[196, 197]
Pamrevlumab (FG‐3019)	Pulmonary fibrosis Pancreatic cancer	Monoclonal antibody therapy	CTGF	Suppresses CAFs activation and ECM deposition.	Phase I/II/III	NCT01262001, NCT00074698, NCT01890265, NCT04419558, NCT03955146	[211, 212, 213]
Calcipotriol	Pancreatic cancer, fibrosis	Vitamin D receptor activation	VDR	Reverses CAF activation and enhances chemotherapy sensitivity.	Phase I	NCT03307148	[214]
Sunitinib	Renal cell carcinoma (RCC)	Multitarget TKI	VEGFR/PDGFR	Inhibits angiogenesis‐related kinases	Phase II	NCT00979966	[215]
Bevacizumab	Metastatic colorectal cancer (mCRC)	Monoclonal antibody therapy	VEGF‐A	Neutralizes VEGF to block angiogenesis.	Phase I	NCT02069704	[216]
LNA‐043	Osteoarthritis	Protein recombinant (angiopoietin‐related protein‐3 stimulator)	Angiopoietin Like 3(ANGPTL3)/Integrin α5β1	Binds integrinα5β1, mimicking fibronectin (FN1) engagement. Upregulates extracellular WNT inhibitors (DKK1, FRZB). Reduces OA‐related gene expression (MMPs, OPN). Promotes hyaline cartilage repair and ECM homeostasis.	Phase II/Phase I	NCT04864392/ NCT02491281	[217, 218]
Sorafenib	NSCLC	Multitargeted angiogenesis inhibition	Raf/MEK/ERK Pathway	Inhibits various kinases involved in angiogenesis, reducing tumor growth and metastasis.	Phase II	NCT00101413 NCT00098254	[219]
Azeliragon	Alzheimer's disease triple‐negative breast cancer	small molecule inhibitor of RAGE	Receptor for Advanced Glycation Endproducts (RAGE)	Inhibits RAGE signaling, reduces Aβ plaque deposition, decreases neuroinflammation, and slows cognitive decline.	Phase II/ Phase III	NCT03980730	[220, 221]

Abbreviations: N/A, Not applicable; NCT Number, National Clinical Trial Number.

### 7.2 ECM Targeted Therapy Strategy Based on Nanotechnology

In recent years, small molecule drugs targeting ECM have become a key strategy to reverse the pathological microenvironment due to their high specificity, regulability, and penetration [222]. Small molecule drugs achieve ECM remodeling by inhibiting ECM‐degrading enzymes and regulating matrix generation signaling pathways. In recent years, ECM‐targeted therapy drugs based on nanotechnology are partially summarized in Table 2. However, due to the inherent hydrophobicity and solid‐state characteristics of small molecule drugs in their chemical structure, they are difficult to tolerate in water, and the tissue penetration efficiency is not high [223]. Nanomedicines have significantly improved the defects of traditional small molecule hydrophobic drugs through unique physical and chemical properties and functionalized design, and have achieved breakthrough progress in multimodal synergistic therapy [224]. Nanodrug‐loaded systems (such as liposomes, polymer micelles, mesoporous silica, etc.) wrap drugs through hydrophobic cores, and the hydrophilic shell forms a stable dispersion system, which significantly improves the apparent solubility of hydrophobic drugs [225, 226, 227]. For example, paclitaxel is more than 1000 times more soluble when encapsulated in liposomes or albumin nanoparticles [228, 229].

**TABLE 2:** Summary of preclinical research on ECM‐targeting drugs.

Agents	Disease Area	Therapeutic Approach	Target	Mechanism	Clinical Stage	NCT Number	Refs
TMP@Alg‐PBA/PVA	Intervertebral disc disease (IVDD)	ECM inhibition	MMP‐3, MMP‐13, ADAMTS‐5	Reduces expression of ECM degradation enzymes, preventing ECM breakdown.	Preclinical	N/A	[230]
HA‐DOX@GNPs‐Met@HFn	Solid tumors	ECM‐depleting and size‐transformable nanodrug	MMP‐2/Tumor cells/CAFs (α‐SMA, Collagen I)	ECM depletion and size reduction improve drug penetration and tumor accumulation.	Preclinical	N/A	[231]
ECM‐drug Conjugates	Cancer	Tumor accumulation and efficacy	ECM	Enhances tumor accumulation and therapeutic efficacy while reducing cardiotoxicity compared with free drugs.	Preclinical	N/A	[232]
HA‐NPs/SMV CV‐NPs/siCol1α1	Hepatic fibrosis	Enzyme therapy	CD44/ECM collagen	Degrades collagen I, enhances ECM penetration, and inhibits HSC activation and autophagy.	Preclinical	N/A	[233]
IONPs‐HAase Nanocarrier	Cancer treatment	Tumor‐targeted drug delivery	Hyaluronic acid (HA)	Breaks down ECM barriers, improving drug delivery and cancer therapeutic efficacy.	Preclinical	N/A	[234]
Alginate Hydrogel	Kidney organoid fibrosis	Biomaterial modulation	Collagen Iα1	Reduces abnormal collagen Iα1 expression via 3D hydrogel encapsulation.	Preclinical	N/A	[235]
Traumatic Acid (TA)	Brain injury	Small molecule inhibition	LOX	Novel LOX inhibitor alleviates neuronal ferroptosis.	Preclinical	N/A	[202]
LR‐SSVA Micelles	Pancreatic fibrosis	ROS‐responsive micelles	Pancreatic stellate cells (PSCs)/LOXL1	Delivers siLOXL1 and resveratrol to reduce ECM crosslinking and ROS‐induced activation.	Preclinical	N/A	[236]
Bruceine D	Breast cancer	Trop2 inhibition	Trop2/β‐catenin	Disrupts Trop2‐β‐catenin positive feedback loop, inhibits EMT and ECM remodeling.	Preclinical	N/A	[203]
RORγ inhibitor	HCC	Nuclear receptor modulation	RORγ	Suppresses matrisome program and ECM remodeling.	Preclinical	N/A	[237]
ERRγ Antagonists	Small‐cell lung cancer (SCLC)	Small‐molecule inhibition	ERRγ	Uppresses ECM remodeling, collagen production, and cell adhesion to inhibit metastasis.	Preclinical	N/A	[238]
dNAc	Breast cancer	NIR‐II‐activated nanotherapy	STING pathway/ECM	Degrades ECM, triggers immunogenic cell death (ICD), and activates the STING pathway.	Preclinical	N/A	[239]
Pirfenidone (PFD)/Doxorubicin (DOX)	Triple‐negative breast cancer (TNBC)	Membrane fusion liposomes(TAFsomes and CCMsomes) sequentially deliver antifibrotic and chemotherapeutic drugs	Tumor‐associated fibroblasts (TAFs)/tumor Cells	PFD inhibits profibrotic cytokines and suppresses collagen synthesis. DOX inhibits DNA replication and transcription, inducing apoptosis in cancer cells.	Preclinical	N/A	[240]

Abbreviations: N/A, not applicable; NCT number, National Clinical Trial Number.

Covalent conjugation of ECM components (such as collagen‐binding peptides) to chemotherapeutic drugs can improve tumor targeting and reduce cardiotoxicity. For example, tumor‐targeting peptide c (RGDyK) modified iron oxide nanoparticles (IONPs) combined with hyaluronidase (HAase) can degrade the tumor matrix, enhance drug penetration, and significantly improve efficacy [234]. Based on the high expression of MMP‐2 in tumor ECM, the MMP‐2‐sensitive nanoparticles designed by researchers can achieve specific drug release at the lesion site. Such systems provide a high‐precision strategy for the treatment of solid tumors through the cascade effect of “enzyme response‐ECM degradation‐drug controlled release”, considering targeted delivery and microenvironment regulation [241].

Beyond enzyme‐driven strategies, nanocarriers can be further engineered to incorporate photosensitizers (PSs) for photothermal/photodynamic ECM degradation, offering spatiotemporal control over therapeutic action. For example, gold nanocages (AuNCs) loaded with indocyanine green (ICG) generate localized hyperthermia under near‐infrared (NIR) irradiation, inducing thermal denaturation of collagen fibers and HA depolymerization. This approach not only softens the ECM but also triggers the release of co‐encapsulated chemotherapeutics (e.g., doxorubicin), achieving dual ECM degradation and drug penetration enhancement [242]. The application of additional ECM‐degrading nano‐strategies, such as ultrasound‐responsive nanodroplets loaded with perfluorocarbon, has been demonstrated to generate cavitation effects, thereby mechanically disrupting the ECM barriers and enhancing nanoparticle accumulation in glioblastoma [243, 244]. These multimodal systems illustrate the convergence of material science, biomechanics, and molecular biology, providing a framework for next‐generation nanotherapeutics that address both cellular and stromal barriers in solid tumors.

### 7.3 Therapeutic Strategies Targeting CAFs

CAFs and the ECM play crucial roles in tumor progression and drug resistance. CAFs contribute to ECM remodeling, cytokine secretion, and epithelial mesenchymal transition, supporting cancer growth and metastasis [245]. In addition, CAFs promote tumor growth and invasion by secreting several cytokines, exosomes, and growth factors, such as leukemia inhibitory factor (LIF) and growth differentiation factor 15 (GDF15) [246, 247]. Based on these data, several studies have focused on targeting CAFs for anticancer therapy.

Firstly, interfering with CAFs activation is an effective strategy, such as inhibiting fibroblast activation protein (FAP) [248]. FAP, a membrane‐bound serine protease overexpressed in CAFs, degrades antifibrotic peptides and remodels the ECM via collagenolytic activity [249]. Genetically engineered T cells expressing FAP‐specific chimeric antigen receptors (CARs) deplete CAFs in desmoplastic tumors [250]. Talabostat (PT‐100) inhibits FAP protease activity, reducing collagen deposition and improving drug delivery [251]. Second, interfering with CAFs signaling pathways is also an important approach, as CAFs activation and function are driven by various signaling pathways, including Hedgehog, NF‐κB, CXCR4, FGFR, and TGF‐β [252]. Therefore, specific inhibitors targeting these pathways are currently being investigated in clinical trials. Finally, promoting CAFs normalization is another viable strategy, which can be achieved through TGF‐β inhibitors or vitamin D analogs like paricalcitol. For example, vitamin D receptor (VDR) activation by paricalcitol downregulates TGF‐β/Smad3 signaling, reprogramming CAFs into quiescent fibroblasts [253].

## 8 Future Perspectives

In contrast to traditional research perspectives, ECM is no longer solely responsible for scaffolding functions. As organisms develop and age, the ECM continues to remodel and plays an important role in pathological processes such as tumorigenesis, fibrosis, cardiovascular disease, and neurodegenerative diseases. However, the potential regulatory mechanisms of the ECM and its key roles in these diseases are not fully understood, the therapeutic effects of targeting the ECM are still not significant, and there are currently few clinically approved ECM‐targeted therapeutics. Therefore, to address the limitations in the current study, future research should further explore the three aspects of experimental modeling, drug development, and therapeutic strategies.

First, there should be a transition from simplified 2D cell culture and single gene‐modified animal models to pathomimetic systems. Existing studies mostly rely on 2D cell culture and animal models, which are difficult to simulate the complex mechanical microenvironment of human ECM, such as stiffness gradient and viscoelastic dynamics changes and other physiological features. Future studies could mimic the mechanical properties of the ECM in fibrotic, neoplastic, or degenerative diseases, such as progressive sclerosis during hepatic fibrosis, by constructing pathology‐specific 3D organoids incorporating patient‐derived fibroblasts and biomaterials such as dynamically crosslinked hydrogels. In addition, by comparing ECM‐related pathways, such as the integrin‐FAK pathway, in different species, we can uncover novel targets for further research and therapeutic development.

Second, research should gradually transition from single‐target therapy to a multitarget strategy of mechano‐chemical co‐regulation. Existing ECM‐targeted drugs (e.g., MMP inhibitors, LOX antagonists) face limitations of off‐target effects or compensatory mechanisms in clinical applications, so innovative design strategies are urgently needed. Inhibitors with dual functions can be developed in the future by designing multitargeted small molecule drugs targeting cross‐regulatory nodes of ECM‐degrading enzymes (e.g., ADAMTS‐5) and mechanistic signaling pathways (e.g., Piezo1/YAP). In addition, AI‐driven ECM ligand screening, combined with deep learning techniques, should be utilized to predict the binding modes of ECM components and receptors, thereby accelerating the optimization and development of lead compounds.

Finally, personalized stratified treatment plans should be developed based on ECM characteristic profiles (e.g., collagen cross‐linking degree, fiber orientation, etc.) of patients. For example, for “high stiffness” tumors, LOX inhibitors can be preferentially used in combination with radiotherapy for sensitization to improve the therapeutic effect. Through the above multidimensional innovative research ideas, we can further promote the clinical application of ECM‐targeted therapy and provide a theoretical basis and practical guidance for the precise treatment of related diseases.

## Author Contributions

Tian Zhao contributed to writing and conceiving the manuscript. Ye Huang drew the figures. Jingfei Zhu, Yujie Qin, Hao Wu, Jiaxuan Yu, and Qianwen Zhai searched and collected literature. Shun Li, Xiang Qin, and Dengfeng Wang revised the manuscript. Tingting Li and Yiyao Liu conceived and revised the manuscript. All authors read and approved the final manuscript.

## Ethics Statement

The authors have nothing to report.

## Conflicts of Interest

The authors declare no conflicts of interest.

## Data Availability

The authors have nothing to report.
